# Farming on the fringe: Shallow groundwater dynamics and irrigation scheduling for maize and wheat in Bangladesh’s coastal delta

**DOI:** 10.1016/j.fcr.2019.04.007

**Published:** 2019-06-01

**Authors:** Urs Schulthess, Zia Uddin Ahmed, Sreejith Aravindakshan, Golam Morshed Rokon, A.S.M. Alanuzzaman Kurishi, Timothy J. Krupnik

**Affiliations:** a CIMMYT-Henan Collaborative Innovation Center, Henan Agricultural University, Zhengzhou, 450002, PR China; b International Maize and Wheat Improvement Center (CIMMYT) and MAIZE CGIAR Research Program, House-10/B, Road-53, Gulshan-2, Dhaka, 1212, Bangladesh; c Research and Education in Energy, Environment and Water. 12 Cooke Hall. University at Buffalo, North Campus, Buffalo, NY, USA; d Farming Systems Ecology Group, Wageningen University and Research, Droevendaalsesteeg 1, Wageningen, 6708PB, the Netherlands

**Keywords:** Water table, Shallow groundwater, Irrigation, Profitability, Soil salinity, Water salinity

## Abstract

Further efforts are needed to combat poverty and agricultural productivity problems in the delta region of Bangladesh. Sustainable intensification of crop production through irrigation and production of cash crops such as maize and wheat might be a promising option to increase income and diversify food production. Only limited research has however been conducted on the potential of using surface water from canals as an irrigation source for maize and wheat production in the delta region. To better understand the contribution of shallow groundwater to crop production and number of irrigations needed for maize and wheat in this unique coastal environment, we conducted multi-locational trials on farmers' fields over three *rabi* seasons. In addition to soil moisture and salinity, we recorded the depth and salinity of the shallow water table throughout these experiments. Maize in particular requires considerable capital investment for seeds, fertilizer, irrigation and labor. Although farmers express wide interest in maize – which can be sold as a profitable cash crop into Bangladesh's expanding poultry feed industry – many of them are reluctant to invest in fertilizer because of the high entry costs. We therefore also investigated the profitability of growing maize under low and high (recommended) fertilizer regimens. Volumetric soil moisture at sowing and during the grain filling phase or at maturity indicated that there is ample supply of water in the profile. Most measurements were above the drained upper limit (DUL). We attributed this to the generally shallow water table depths, which never exceeded 2.75 m at any location, but generally stayed between 1–2 m depth throughout the season. The region’s soil texture classes (clay loams, silt loams and silty clay loams) are all conducive for capillary rise of water into the rooting zone. Consequently, irrigation had a significant effect on maize yield in the driest winter only, whereas for wheat, it had no effect. The key for a successful maize and wheat production in the delta region of Bangladesh is to ensure a good crop establishment, which can be achieved with a starter and an additional irrigation at crown root initiation for wheat and at V6-8 for maize. Maize however is not always profitable. Compared to low fertilizer rates, higher rates reduced losses in low yielding site-years and increased profits in high-yielding site years. This indicates that it is advisable for farmers not to reduce fertilizer rates. Low-risk financial credit with rationally structured interest rates that allow farmers to invest in maize could potentially offset these constraints.

## Highlights

• Maize and wheat are proposed for diversification, but irrigation advice is limited.
• Soil moisture content was above or near drained upper limit for depths below 0.5 m.
• A significant effect of irrigation on yield was observed for maize only, in one year.
• One irrigation, in addition to a starter irrigation, generally is sufficient to grow a crop.
• Low-input maize cultivation is not always profitable and low fertilizer rates increase risk of negative returns.

## 1 Introduction

Over the last two decades, Bangladesh has greatly increased production of rice, its staple food. Stunting in children below the age of 5 has decreased from 43% in 2007 to 36% in 2015 (IFPRI, 2015). But further efforts are needed to totally eliminate it. Headey and Hoddinott (2016), for example indicate dietary diversity has remained among the lowest globally, and that delays in the introduction of complementary foods – and most likely, inadequate calorie intake of children - are related to low levels of agricultural productivity, inconsistent income generation, and the low resource-endowments of smallholder households. Consequently, increased public investments in staple food production, in addition to agricultural diversification and linkages to favorable output markets, are widely proposed.

**Fig. 1:**
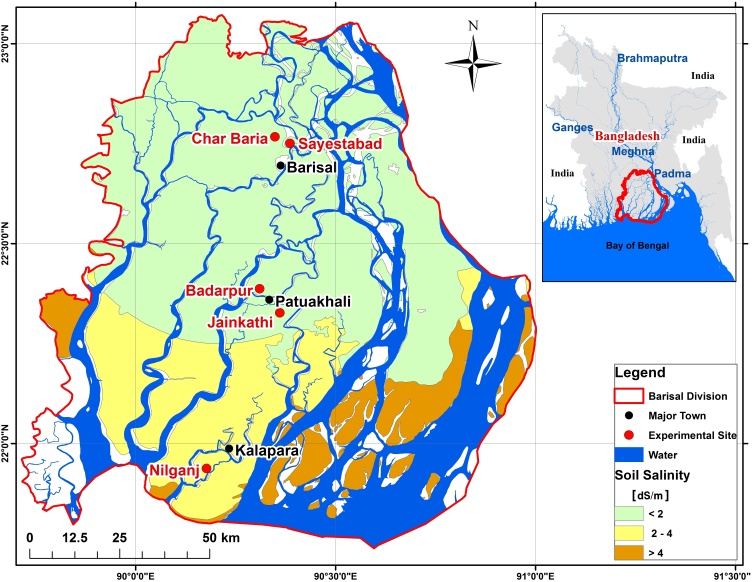
Map of experimental sites. All trials were conducted on farmers' fields in the Barisal Division of Bangladesh during the *rabi* seasons of 2014-15, 2015-16 and 2016-17. Soil salinity data are based on SRDI (2000).

Most of the increase in rice production in Bangladesh stems from *boro* rice in the northern half of the country, where irrigation with water from shallow and deep tube wells permits farmers to grow *boro* rice in the winter *rabi* season as a second crop after summer monsoon *aman* rice (Qureshi et al., 2015). However, contrary to the north, groundwater in the coastal south tends to be saline. Large scale abstraction is not an option. So far, little intensification of crop production has taken place in the Barisal division (Bhattacharya et al., 2019), our study area (Fig. 1). The region is dominated by a low input-output crop production system. Irrigation and fertilization are limited to pockets of *boro* rice, vegetable and fruit production. Agricultural mechanization and irrigation pumps are comparatively rare (Mottaleb et al., 2016). Farmers predominantly grow traditional rice varieties that can withstand deep flooding during the summer monsoon. Key winter *rabi* season crops include grass pea (*Lathyrus sativus* L.) and mung bean (*Vigna radiata* (L.) Wilczek). Both are grown with little nutrient management or irrigation, their productivity generally depends on favorable weather conditions. Focusing on Barisal Division, Krupnik et al. (2017) reported that 390,000 ha are either regularly fallowed or under low production intensity during the winter. The same study, as well as Bhattacharya et al. (2019) indicated considerable potential to increase cereal production and revenue using available surface water for irrigation. The region however also experiences other biophysical constraints. Severe local convective storms peak in April (Yamane et al., 2010a, 2010b). They can cause crop lodging and short duration waterlogging.

Barisal Division covers 13,225 km and is mostly flat, ranging from 2 to 4 m above sea level. Numerous rivers and naturally flowing canals meander through the division. Irrigation using efficient pumps to lift surface water and convey it to fields might be a viable option (Krupnik et al., 2015a). The depth of the water table in this region ranges between 0.5 to 3 m (Ahmed, 2011; Mainuddin et al., 2014). This implies that the water table is close to the surface and crops might be able to tap into the capillary fringe, lowering irrigation requirements and increasing the cost-effectiveness of crop production. However, high levels of soil water salinity (SRDI, 2000) may pose a risk for the crops, especially in the southern half the study area.

Studies by Dalgliesh and Poulton (2011) and Mainuddin et al. (2014) indicated the potential for efficient production of wheat because of the contribution of groundwater to crop growth in the coastal zone. Apart from those reports, we are unware of any previous studies that have measured the impact of shallow groundwater availability and salinity dynamics on irrigation requirements at the field level in coastal Bangladesh. The introduction of alternative cereal crops, including wheat and maize, will also require careful nutrient alongside water management. We therefore also investigated the profitability of maize production under low and recommended nutrient regimes, in order to develop economically viable recommendations for farmers.

## 2 Material and methods

On-farm experiments were conducted during the winter (*rabi*) seasons at three locations within the Barisal Division (Table 1). At two locations, Barisal and Patuakhali, we shifted to new sites after the first year to accommodate farmers willing to lend fields for experiments over multiple seasons. Maize was grown in three seasons (2014/15, 2015-16 and 2016-17), while wheat was grown in the first two seasons only, because of an outbreak of wheat blast (*Magnaporthe oryzae* pathotype Triticum) in early March of 2016 (Malaker et al., 2016), after which Bangladesh’s Department of Agricultural Extension recommended farmers reduce wheat cultivation in the subsequent 2016-17 season in the affected areas. All experiments were conducted on farmers' fields given available land area, on which Transplanted (T.) *aman* rice had been grown during the prior monsoon seasons. Rice was harvested in December with wheat and maize sown when fields became trafficable after the recession of flood water. Trials were researcher designed and implemented with the cooperation of farmers. Plot sizes were 20 by 25 m in the first, 8 by 15 m in the second and 10.8 by 10 m in the third year. We planted the maize varieties NK-40 in year one and Pioneer P3396 in the following years as per farmers’ preferences. Row spacing was 0.6 m and plant to plant distance 0.2 m. For wheat, BARI GOM 26 was sown in both years of experimentation, at a seed rate of 120 kg ha^−1^. A detailed characterization of the soil properties can be found in Annex A.

**Table 1:** Experimental locations, geographic coordinates and elevations of farmers' fields on which the trials were conducted during the 2014/15–2016/17 winter *rabi* seasons.

Location	Site (village)	Rabi season(s)	Coordinates (Latitude – Longitude)	Elevation (m.a.s.l.)
Barisal	Char Baria	2014/15	22.7675 – 90.3449	2.4
	Sayestabad	2015/16; 2016/17	22.7515 – 90.3859	1.7
Patuakhali	Badarpur	2015/16; 2016/17	22.3890 – 90.3074	3.3
	Jainkathi	2014/15	22.3285 – 90.3610	3.2
Kalapara	Nilganj	2014/15; 2015-16; 2016-17	21.9377 – 90.1771	2.3

### 2.1 Irrigation treatments

The main objective of these experiments was to determine locally appropriate irrigation schedules for dry winter *rabi* season maize and wheat production. For all treatments in this study, low-lift pumps were used to draw water from natural canals for irrigation (see Krupnik et al. (2015a) and Krupnik et al. (2017) for description of the region’s irrigation systems). Two separate completely randomized block experiments, with three replications each for maize and wheat, were conducted. All treatments had received a uniform starter irrigation at sowing.

For maize, three irrigation treatments were applied. (1) a ‘dry’ treatment with one irrigation applied at vegetative (V) stage 8 (V8), (2) an ‘intermediate’ irrigation treatment with irrigation applied at V8 and tasseling, and (3) a ‘wet’ irrigation treatment, with irrigation applied at V8, tasseling, and soft dough. For wheat, treatments included (1) a dry treatment with irrigation applied at crown-root initiation (CRI), (2) an ‘intermediate’ treatment with irrigation applied at CRI and 50% heading, and (3) a ‘wet’ treatment with irrigation applied at CRI, 50% heading, and the early milking stage. Averaged across sites and years, total irrigation amounts for the dry, intermediate and wet treatments were 126 mm, 227 mm and 278 mm for maize, and 88 mm, 178 mm and 223 mm for wheat, respectively.

### 2.2 Fertilizer treatments

As a high-yielding and biomass producing crop, maize yields depend greatly on nutrient availability (Connor et al., 2011). In smallholder dominated farming systems such as those found in Bangladesh’s deltaic region, farmers do not always have the financial means to purchase large amounts of fertilizer (Krupnik et al., 2017, 2015b). We therefore investigated the impact of low vs high fertilizer (Low Fertilizer and High Fertilizer) rates on crop and economic productivity of maize. The two fertilizer treatments (rates described below) were subjected to the three irrigation treatments described above during the 2015-16 and 2016-17 seasons. Please note that only yield data from the High Fertilizer plots were used when reporting the effects of irrigation on yield.

### 2.3 Crop management

Wheat was sown in the second half of December, and maize around January 1, except for the Jainkathi site in 2015, where sowing could be accomplished by January 19 only. The experiments were planted on raised beds alternating with furrows (through which surface irrigation can be channeled) at Barisal and Patuakhali in the first 2 years. In the 3rd year, strip tillage and subsequent earthing-up at V8 was used. Prior to bed-planting, the soil was prepared with two power tiller passes, except for the 2014/15 Patuakhali maize site, which received one pass only. Bed and strip-tillage planting followed the configurations detailed by Gathala et al. (2015) and Gathala et al. (2016) using equipment similar to that described by Krupnik et al. (2013). The only herbicide application was made for the strip-tilled maize fields in 2016/17. Glycel (Glyphosate) at a dose of 3.7 l ha^−1^ was applied prior to planting. Otherwise, weeds were controlled manually. In maize, cut worms were controlled with one application of Karate (Lambda-cyhalothrin) at 0.75 l ha^-1^. A mixture of Belt (Flubendiamide) at 205 g ha^–1^ and Decis (Deltamethrin) at 0.494 l ha^–1^ was used against cob borer. Rats infested the wheat plots only and were controlled as required.

Fertilization was done according to national recommendations from BARC (Chowdhury, 2013). For wheat, the rates were: Urea (101.2 kg N ha^−1^); Triple Super phosphate (TSP) (36 kg P ha^−1^); Muriate of Potash (MOP) (25 kg K ha^−1^), gypsum (21.6 kg S ha^−1^). For maize, the rates for the High Fertilizer treatments were: Urea (255 kg N ha^−1^); TSP (56 kg P ha^−1^); MOP (125 kg K ha^−1^), Gypsum (21.6 kg S ha^−1^), zinc sulphate (5.4 kg Z ha^−1^) and borax (1.4 kg B ha^−1^). The Low Fertilizer treatment was based on an informal survey conducted with 10–12 farmers at each location. We asked them how much fertilizer they would typically be able to purchase to grow a maize crop. Their average rates, which amounted to roughly half of the official rates, are listed in the footnote of Table 5. Nitrogen applications were split thrice for the medium and wet irrigation treatments. For the dry treatment, for which only one in-season irrigation was foreseen, 1/3 of the N was applied at sowing and the remainder just before the first irrigation.

The site at Kalapara has high salinity, both in the soil and ground water. A full irrigation at sowing would likely have caused a rise of the water table and subsequent salinization of the seed bed. For seeding and the starter irrigation, we therefore relied on an established method developed by the farmer whose fields were used for the experiments. He prepares the seedbeds with two power tiller passes, and then plants the crop with a power tiller operated seeder. Immediately after sowing, he then applies a "shower" irrigation. The irrigation amount is about 10–15 mm, just enough to ensure proper germination. Wheat took about 100 and maize 150 days to reach maturity, when plots were harvested to measure grain yield.

### 2.4 Measurements

#### 2.4.1 Irrigation rates and salinity

The amount of water applied for each irrigation was measured with a flow meter and salinity with a CTD-Diver (Van Essen Instruments B.V) that was used for the piezometers as well (see 2.4.4).

#### 2.4.2 Soils

At each site, we excavated three soil profiles, 1 m deep. This was done just prior to sowing of the first crop in year one and in early February of 2016 for the two new sites that were used in the second year. Samples were collected at depth increments of 0.1 m. In addition, intact cores were taken from each depth to determine bulk density. The Soil Research Development Institute (SRDI) of Bangladesh analyzed the following soil parameters for each sample: texture, organic carbon and pH according to methods described in SRDI (2014). Average soil properties for each site are described in Appendix A. Across locations, soils were predominantly clay loam, silt loam and silty clay loam.

We estimated volumetric soil moisture content at sowing and once during grain filling or just after harvest, weather (flood) conditions permitting. Sampling depth was 1 m, with a depth interval of 0.1 m. For the first year, samples from the soil profiles were used to estimate initial moisture conditions. All the other samples for soil moisture were gathered from the center of each plot. One portion of these samples was set aside for a separate soil salinity analysis and from the other portion, we took the fresh weight and dry weight, after drying the samples in the oven at 105 °C for 72 h. Based on these weights, we then calculated volumetric soil moisture content, taking into account bulk density as well. We used the functions reported by Ritchie et al. (1999) to calculate the lower limits (LL) and drained upper limit (DUL) for each profile. For the calculation of the saturated volumetric soil water content (SAT), the model assumes a particle density of 2.65 Mg m^−3^ and porosity was multiplied by a factor of 0.92, in order to correct for the fraction of entrapped air, which was assumed to be 0.08.

According to SRDI (2000), soil salinity at the most southern location is between 2–4 dS m^−1^, while at the other sites, it is below 2 dS m^−1^ (Fig. 1). Salinity levels may change due to leaching caused by irrigation or rain, as well us due to upflow from lower depths. We analyzed soil salinity for the plots at Kalapara in all three years and at Patuakhali in the second year. At Barisal, where very low salinity levels were expected, we only analyzed data from the wheat crop for the 2015/16 season. From the 2014-15 experiment at Kalapara, samples had been taken to a depth of 0.5 m only at sowing. At all other dates, soil was sampled from the center of each plot down to 1 m. For all dates, depth increments were 0.1 m. Soil salinity was estimated from a saturated soil-paste extract by measuring electrical conductivity as described by Rhoades et al. (1989).

#### 2.4.3 Meteorological measurements

Precipitation data were provided by the Bangladesh Meteorological Department (BMD). The respective stations for the three locations were within eight km or less from the trial sites. Rainfall varied greatly among years (Table 2). 2015 had occasional light rains between January and March, with more intense rains starting in April. 2016 was comparatively dry, especially in March and April. On May 21, just after harvest of the last maize trials, the tropical cyclone *Roanu* made landfall. It brought 400–600 mm of rain in 3 days. In 2017, January and February had no rain, until a tropical depression resulted in more than 100 mm of rain between March 8–11 at Kalapara and Patuakhali. These rains were followed by more heavy ones in April.

**Table 2:** Total monthly rainfall (mm) measured in the three trial regions as reported by the Bangladesh Metrological Department (BMD) between January and May of 2015–2017. The official name of the BMD station at Kalapara is Khepupara.

	Barisal			Patuakhali			Kalapara		
	2015	2016	2017	2015	2016	2017	2015	2016	2017
January	3	1	0	9	12	0	8	6	0
February	24	31	0	4	53	0	15	13	0
March	18	8	59	33	3	119	18	3	155
April	135	51	365	179	42	250	102	22	109
May	72	438	118	38	454	56	41	582	78

#### 2.4.4 Water table depth and salinity

At each site, we had installed piezometers following methods similar to those described by Bouman et al (2007). Unfortunately, several piezometers were damaged by cattle. All data points reported here are based on seven or more piezometers. At Kalapara and Patuakhali, where the shallow water tables were expected, the piezometers were 3 m deep, while at Barisal, they were installed to 5 m. The top 1 m of the piezometer was solid (impermeable), while porous material was used for the remainder. We used a CTD-Diver (Van Essen Instruments B.V.) to measure the distance from the top of the piezometer to the water table as well as water salinity (at the top of the column) at each field visit.

#### 2.4.5 Yield

Grain yields were determined at physiological maturity after hand harvesting the entire plots. We had also measured yield from multiple sampling locations measuring 1.2 m^−2^. Those yields were consistently higher than the whole plot yields. We therefore report yield from the whole plot as a more conservative measure of productivity. Since our plots measured between 108 and 500 m^2^ with a large surface area to edge ratio, depending on the land size farmers were able to allocate, border effects are likely to have been minimal. Wheat threshing was done with a closed drum thresher and a maize sheller was used to separate the grains from the hand harvested cobs. Two grain samples per plot were taken to determine fresh and, after drying in the oven for 48 h at 70 °C, dry weight of the grains. All reported yield data are moisture corrected (14%).

### 2.5 Computations and analyses

#### 2.5.1 Statistical analysis of irrigation treatments

Separate standard analyses of variance (ANOVAs) were conducted to test for significant irrigation treatment effects across sites and years for maize and wheat. In addition to the irrigation treatments, maize had low and high fertilizer treatments in years two and three in all sites. In order to maintain analytical consistency across all three years, only the high fertilizer plots were considered when testing for irrigation effects.

#### 2.5.2 Economic analysis

Our economic analysis is based on straight forward benefit-cost ratios (BCRs), which can be defined as total capitalized benefits divided by total capitalized cost, both in monetary terms. The total capitalized benefits in our analysis is represented by gross revenue from maize cultivation (GR). The monetary values of maize grain and stover sales from each of the Low and High Fertilizer trial plots during 2016 and 2017 were separately recorded for the computation of gross revenue. For the estimation of total capitalized costs, the cost of all direct expenses incurred for growing maize under low and high fertilizer treatments, land preparation costs, seed, fertilizer, and intercultural management and harvest labor costs are included in the estimation. Apart from the application of Glycel prior to strip-till planting of the 2016/17 maize crop, all weed management was carried out through manual weeding. Weed control and seed rates were more or less uniform across the trial plots, irrespective of fertilization. The imputed values of family labor applied by farmers participating in trial plot management were estimated based on the current wage rates for male and female agricultural labors as observed during the season. These were used in the cost-return analysis. Although cost of production of maize includes both variable costs and fixed costs, in this paper we used variable cost for computing BCR since they vary with local market conditions. They can drive farmers' decisions on input purchase and the ratio of maize sold vs. retained by farming households. We computed *Cost α* (Operating Cost), which involves all variable costs except imputed value of family labor and *Cost β,* which is *Cost α +* imputed value of family labour. The BCR over paid-out cost (BCR_1_) and BCR_2_ (BCR over paid-out + family labor cost) can be computed following Eqs. (1) and (2), respectively:

(1) $$BCR1\;=\frac{GR\;{ha}^{-1}}{Cost\;\alpha \;{ha}^{-1}}$$

(2) $$BCR2\;=\frac{GR\;{ha}^{-1}}{Cost\;\beta \;{ha}^{-1}}$$

In addition, we also estimated the rate of return (RoR) for maize crop from fertilizer investment over a period of two years, expressed as a percentage. This is shown below:

(3) $$\text{R}\text{o}\text{R}\;=\frac{GR\;{ha}^{-1}-\;Cost\;\alpha \;{ha}^{-1}}{Cost\;\alpha \;{ha}^{-1}}\;\times 100$$

A two-way, two-factor ANOVA was also used to test for statistical significance of the effect of nutrient levels and trial locations.

## 3 Results

### 3.1 Water table dynamics

#### 3.1.1 Water table depth

**Fig. 2:**
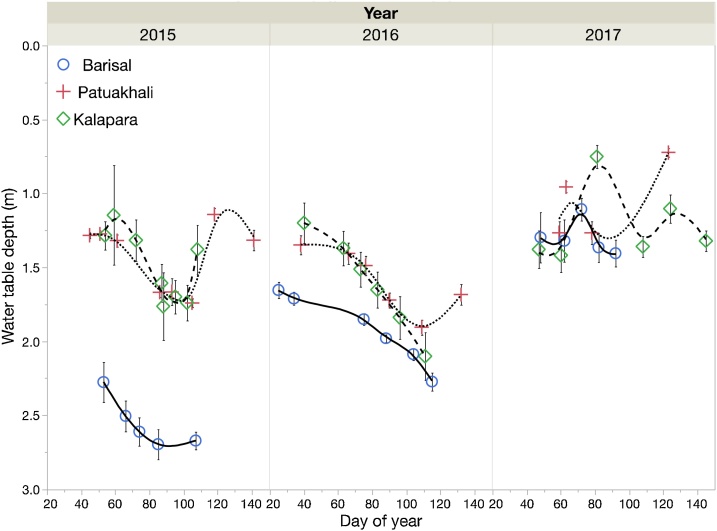
Depth of water table (distance from the ground surface to the top of the water table) across seasons and locations. Each point represents the average depth measured from at least 7 piezometers. Error bars represent one standard error of the mean.

At all three sites, the water tables remained close to the surface throughout each winter *rabi* season (Fig. 2). The deepest water table was measured for the Chandpasha (Barisal) site in 2015, where its depth peaked at 2.75 m. At the other sites and the other years, depth fluctuated mostly between 1 and 2 m. The decline in water table depth during the three *rabi* seasons, calculated as the difference between the observations from the days with the highest and lowest water table observations ranged between 0.62 and 0.76 m at Kalapara, 0.54 and 0.60 m at Patuakhali and 0.3 and 0.62 m at Barisal. Considering periods of prolonged and constant declines, the average daily decline was 0.013 m and 0.007 m at Barisal and 0.014 and 0.011 m at Kalapara, in 2015 and 2016 respectively, while at Patuakhali, it was 0.008 m in both seasons. In 2017, heavy rains between March 8 and 11 caused a remarkable raise of the water table at all 3 sites, and therefore, no prolonged period of decline was observed.

#### 3.1.2 Water table salinity

**Fig. 3:**
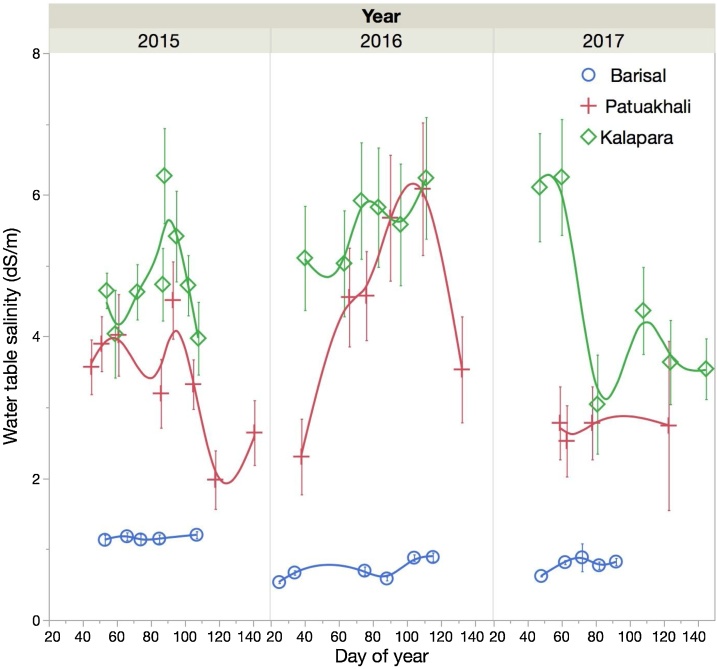
Water table salinity levels measured at the trial sites during the *rabi* seasons across three years and three regions in the Barisal division of Bangladesh. Error bars represent one standard error of the mean. For Barisal and Patuakhali, different sites than in 2015 were used in 2016 and 2017.

Water table salinity (Fig. 3) was low for Char Baria and Sayestabad (Barisal) locations in all years, with values fluctuating around 1 dS m^−1^. At Patuakhali, large variability in salinity was observed: At the Jainkathi site, which was used in the first year, salinity levels varied between 2–4 dS m^−1^. At Badarpur, salinity levels increased up to 6 dS m^−1^ in 2015/16, while they ranged between 2–3 dS m^−1^ in 2016/17. As expected, the highest salinity levels were measured at Kalapara, where they ranged between 4–6 dS m^−1^, although they sharply dropped in March of 2017, after the region had received 155 mm of rain in 4 days. This drop in salinity coincided with a raise of the water table depth by 0.8 m (Fig. 2).

#### 3.1.3 Irrigation water salinity

The salinity of irrigation water drawn from naturally flowing canals varied across locations. At both Barisal sites and at Badarpur, irrigation water salinity never exceeded 1 dS m^−1^. At Jainkathi (2015), it remained below 1 dS m^–1^ until mid-March and peaked at 1.5 dS m^−1^ in mid-April. At Kalapara, which is most proximal to the coast of all experimental sites, a nearby canal with direct connectivity to the ocean serves as main source of irrigation water. Farmers are able to manage water flow into and out of the canal to a limited extent using aged sluicegate infrastructure. They attempt to trap fresher water at the end of the monsoon season by closing the canal, using impounded water for irrigation during the dry winter season. They are however only partially effective in maintaining freshwater quality because of leaks in the sluicegate structure. Large differences were therefore measured among years. In 2014-15, average salinity was 5.9 dS m^–1^, while in 2015-16, it was 3.4 dS m^–1^ and 2.4 dS m^–1^ in 2016-17.

### 3.2 Soil moisture and salinity observations

#### 3.2.1 Soil water content

**Fig. 4:**
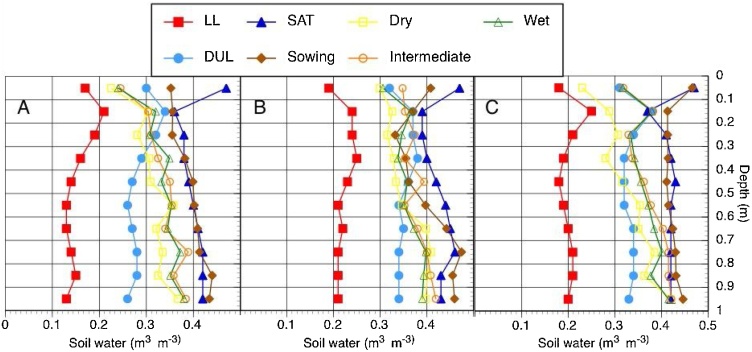
Soil water limits (LL = lower Limit; DUL = Drained Upper Limit; SAT = Saturated), volumetric soil moisture at sowing and during grain filling for three irrigation treatments (Dry, Intermediate, or Wet) of wheat sown at three locations (A = Barisal; B = Patuakhali; C = Kalapara) in Bangladesh in the winter of 2014/2105.

Volumetric soil moisture data for the first two experimental seasons from all sites are shown in the Appendix B. No data are shown for the last season, because high rainfall caused saturated conditions for all treatments at harvest. For all sites, moisture content was above DUL for the top 0.2 m of the profile at sowing and most measurements during the grain filling phase or at harvest were near or above DUL as well. Fig. 4 illustrates the distribution of soil moisture within the profile at sowing and during the grain filling phase for wheat during the 2014/15 season. At Barisal and Kalapara, similar patterns were observed for depths below 0.2 m: At sowing, moisture levels were close to saturation and during grain filling, they were between DUL and saturation, except for the dry treatment at Kalapara, for which moisture levels had dropped below DUL for depths from 0 to 0.4 m. At Patuakhali, all measurements were above 0.3 m^3^ m^−3^, although some of them were below DUL. Among the treatments, a tendency to lower moisture levels was observed for the dry treatment. Maize reached the grain filling phase in April only, after the sites had experienced some rainfall. Moisture levels below DUL were observed for some depths at Patuakhali and Kalapara only.

#### 3.2.2 Soil salinity

**Fig. 5:**
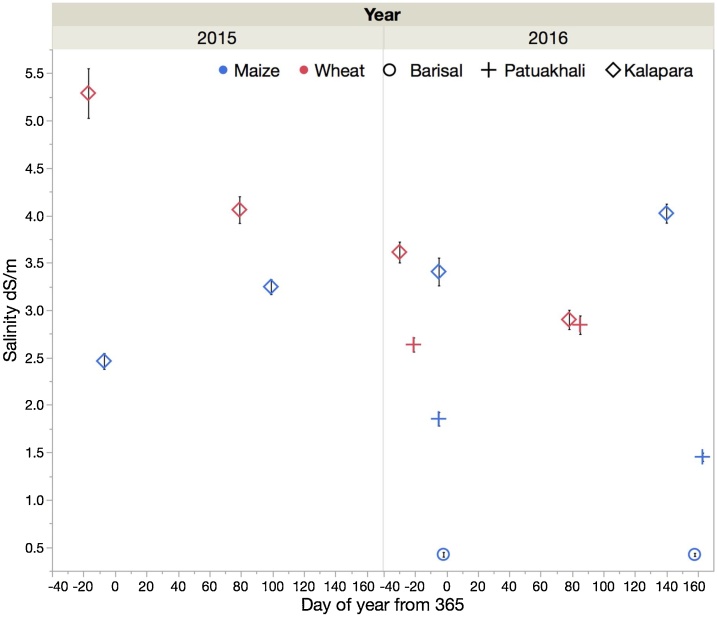
Soil salinity of maize and wheat experiments measured at the three experimental locations in coastal Bangladesh during the 2014-15 and 2015-16 seasons. Samples were taken at 0.1 m increments up to 1 m depth, from the center of each of the 9 plots, except for the data points collected at sowing for the 2015 data reported for Kalapara, where sampling depth was 0.5 m only. Means were calculated based on measurements from the entire profile. Error bars represent the standard error of the mean.

**Fig. 6:**
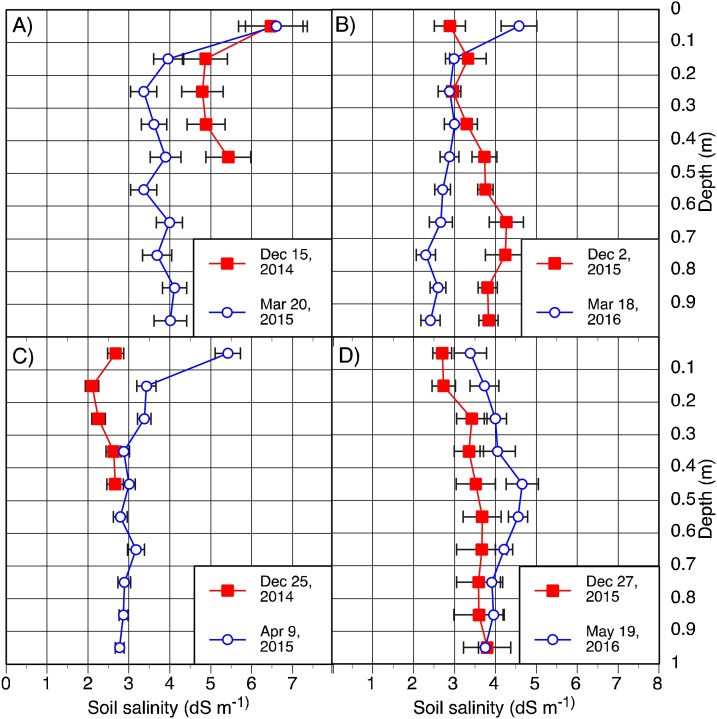
Soil salinity at Kalapara (Bangladesh) for wheat (A, B) and maize (C, D) measured at the beginning and around the end of the growing period. Error bars represent the standard error of the mean.

At Barisal, soil salinity was low, at around 0.5 dS m^−1^, while at Patuakhali, it was just below 3 dS m^−1^ for the wheat and a bit lower for the maize plots in the 2015/16 growing season (Fig. 5). The highest soil salinity levels were observed for the 2014-15 wheat field at Kalapara. A detailed analysis of its vertical distribution revealed that at sowing, the concentration in the top 0.1 m was 6.5 dS m^−1^, 1.6 units higher than for the layers between 0.1 and 0.4 m depth (Fig. 6). This was the only field where soil salinity was higher than 4 dS m^-1^ at sowing. At the time around harvest of wheat, salinity was at least 1.5 dS m^-1^ higher for the top layer (0-0.1 m depth) as compared to the layers below in both years at Kalapara. It measured 6.5 dS m^-1^ in March 2015 and 4.5 dS m^-1^ in March 2016. A similar distribution across the profile was observed for maize in March 2015, whereas in 2016, when sampling took place in May only, no clear trend was observed. At Patuakhali, no such accumulation of salt in the top 0.1 m of the soil could be observed either (data not shown).

### 3.3 Crop yield

#### 3.3.1 Maize yield

**Fig. 7:**
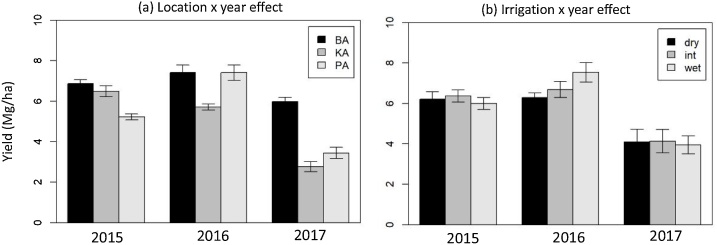
Variability of maize yield among a) experimental sites and years and b) irrigation treatments and years. Each site-year therefore represents the average of the three irrigation treatments. Similarly, each irrigation-year represents the average of three sites. The irrigation treatments were: dry, intermediate (int) and wet. BA stands for Barisal, KA for Kalapara and PA for Patuakhali. Error bars represent the standard error of the mean.

ANOVA results for maize grain yield indicated highly significant (P < 0.001) effects for location and year, as well as a location by year and year by irrigation interaction (Table 3). The highest maize yields (7.41 Mg ha^−1^) were observed at Barisal and Patukhali in 2015/16 (Fig. 7a). Barisal was the highest yielding location in all three years, followed by Patuakhali and Kalapara. Irrespective of sites, the statistically significant highest mean yield was observed for wet irrigation followed by intermediate irrigation in year 2016 (Fig. 7b). However, high irrigation did not perform better than the other two treatments in the first and last year of the experiments. The lowest yield was obtained in all irrigation treatments in 2017, the year in which heavy rains in March caused flooding around V12 growth stage.

**Table 3:** Analysis of variance of the effects of location, year and irrigation on maize and wheat yield grown at three sites in Bangladesh. Irrigation treatments included: low, medium and high.

		Maize yield		Wheat yield
Treatment	DF	*P* > F	DF	*P* > F
Location (LO)	2	0.000**	2	0.641
Year (Y)	2	0.000**	1	0.000**
Irrigation (IR)	2	0.315	2	0.592
LO × Y	4	0.000^*^	2	0.000**
LO × IR	4	0.413	4	0.104
Y × IR	4	0.015^*^	2	0.634
IR × REP	6	0.255	6	0.465
LO × Y × IR	8	0.078	4	0.308

^*^,**,*** Significant at the 0.05, 0.01 and 0.001 probability levels, respectively.

#### 3.3.2 Wheat yield

**Fig. 8:**
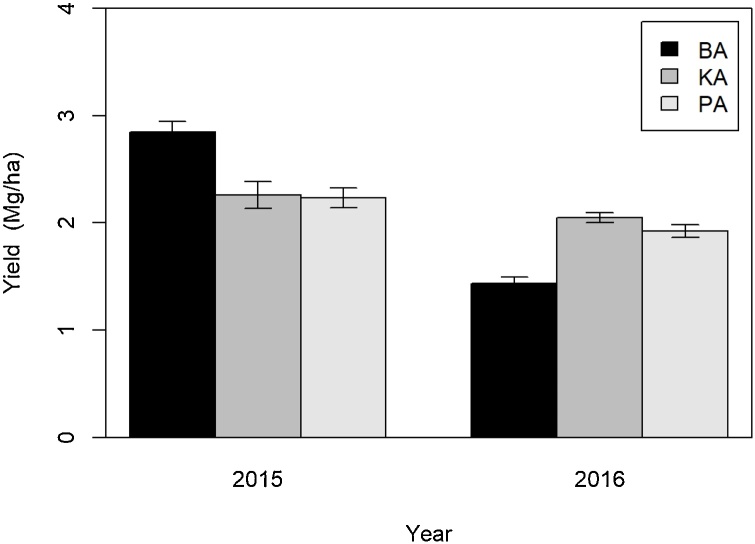
Variability of wheat yield among experimental sites and years. Irrigation treatments had no significant effect on yield. Each site-year therefore represents the average of the three treatments. BA stands for Barisal, KA for Kalapara and PA for Patuakhali. Error bars represent the standard error the mean.

Wheat was grown in the first two winter seasons only. An outbreak of wheat blast and *Helminthosporium* spp. caused a collapse of the green canopy within 2–3 days, and significant yield reductions in the 2015/16 season. Barisal was most affected by the disease. Yield levels at Patuakhali and Kalapara were similar in both years (Fig. 8). Irrigation had no significant effect on yield in wheat.

### 3.4 Maize yield and economic performance under high and low fertilizer rates

For the economic profitability assessment, we implemented two fertilizer rates, low (Low Fertilizer) and high (High Fertilizer) in 2015-16 and 2016/17. We used a two-way, two-factor ANOVA to test for statistical significance of the effect of fertilizer levels and trial locations (Table 4). There was a significant and positive effect for fertilizer level on maize yields in both years. The effect of location as well as the interaction effect between location and fertilizer was also found to be significant. Unlike 2015-16, the effect of location however was large in 2016-17, possibly due to a wide range of yield across different trial locations (i.e. a maximum of 6.73 tons ha^−1^ at Barisal and 0.5 tons ha^−1^ at Kalapara). The interaction effect of fertilizer and location was also positive and significant. Fertilization treatment had a significant effect at all sites (P < 0.001).

**Table 4:** Analysis of variance of the effects of fertilizer and location on maize yield across two years and three sites in coastal Bangladesh. Fertilizer treatments included: high (official recommendation) and low (according to farmer's willingness to invest in fertilizer).

		2015/16			2016/17	
Treatment	DF	*P* > F		DF	*P* > F	
Fertilizer (FE)	1	0.000	***	1	0.000	***
Location (LO)	2	0.010	**	2	0.000	***
FE × LO	1	0.010	**	2	0.010	**

^**^,*** Significant at the 0.01 and 0.001 probability levels, respectively.

**Table 5:** Economics of maize cultivation under low (LF) and high fertilizer (HF) rates (in USD ha^−1^) during 2 seasons at 3 locations in the coastal delta of Bangladesh.

Costs and return	Cost	Barisal (*n = 9*)				Patuakhali (*n = 9*)				Kalapara (*n = 9*)			
		Year 2016		Year 2017		Year 2016		Year 2017		Year 2016		Year 2017	
		LF^a)^	HF	LF ^a)^	HF	LF^b)^	HF	LF ^b)^	HF	LF^c)^	HF	LF ^c)^	HF
Seed	5.09 USD 20 kg bag^−1^	101.76	101.76	101.76	101.76	101.76	101.76	101.76	101.76	101.76	101.76	101.76	101.76
Inorganic fertilizers													
*Urea*	0.20 USD kg^−1^	48.35	108.60	48.35	108.60	50.11	108.60	50.11	108.60	50.25	108.60	50.25	108.60
*Triple Super Phosphate*	0.28 USD kg^−1^	41.97	74.48	41.97	74.48	40.57	74.48	40.57	74.48	50.37	74.48	50.37	74.48
*Muriate of Potash*	0.19 USD kg^−1^	17.93	38.00	17.93	38.00	14.12	38.00	14.12	38.00	21.37	38.00	21.37	38.00
*Gypsum*	0.29 USD kg^−1^	23.40	60.32	23.40	60.32	18.14	60.32	18.14	60.32	13.16	60.32	13.16	60.32
*Zinc Sulphate*	2.04 USD kg^−1^	0.00	28.34	0.00	28.34	0.00	28.34	0.00	28.34	0.00	28.34	0.00	28.34
*Boron as Boric acid*	2.80 USD kg^−1^	0.00	14.00	0.00	14.00	0.00	14.00	0.00	14.00	0.00	14.00	0.00	14.00
Pesticides and herbicides													
*Herbicide*^d^	7.25 USD ltr^−1^	23.66	23.66	16.54	16.54	23.66	23.66	16.54	16.54	23.66	23.66	16.54	16.54
*Cut worm control*^e^	USD 12.52 ltr^−1^	10.16	10.16	10.16	10.16	10.16	10.16	10.16	10.16	10.16	10.16	10.16	10.16
*Cob borer control*	USD 12 for 100 g Belt + USD 1.65 per 100 ml Decis	32.94	32.94	32.94	32.94	32.94	32.94	32.94	32.94	32.94	32.94	32.94	32.94
Machinery use for land preparation and sowing													
*Machine hiring + operation*	a. 34.91 USD ha^−1^ per tillage pass using a power tiller’ b. 78.70 USD ha^−1^ per strip tillage establishment	104.73	104.73	104.73	78.70	104.73	104.73	104.73	78.70	104.73	104.73	104.73	104.73
Irrigation service (pump hire + fuel)	0.51 USD hr^−1^	97.51	97.51	97.51	97.51	97.51	97.51	97.51	97.51	97.51	97.51	97.51	97.51
Labor													
*Hired labor*	5.08 USD psd^−1^	254.39	254.39	254.39	254.39	254.39	254.39	254.39	254.39	254.39	254.39	254.39	254.39
*Opportunity cost of family labor (imputed)*^f^	5.08 USD psd^−1^	183.16	183.16	183.16	183.16	183.16	183.16	183.16	183.16	183.16	183.16	183.16	183.16
Total cost of cultivation													
*Paid-out* (USD ha − 1)	−	756.80	948.89	749.68	915.74	748.09	948.89	740.97	915.74	760.30	948.89	753.18	941.77
*Paid-out + opportunity cost of family labor* (USD ha − 1)	−	939.96	1132.05	932.84	1098.90	931.25	1132.05	924.13	1098.90	943.46	1132.05	936.34	1124.93
Maize grain yield (Mg ha − 1)	0.23-0.25 USD kg^−1^	4.64	7.41	2.77	5.98	4.53	7.41	1.70	3.44	4.58	5.71	1.09	2.77
Maize stover yield (Mg ha − 1)	0.013 USD kg^−1^	5.44	8.74	3.35	7.06	5.31	8.37	2.08	4.10	5.04	6.97	1.34	3.38
Gross revenue (USD ha − 1)	−	1249.97	1995.24	723.11	1559.77	1219.38	1990.21	445.02	897.94	1113.45	1540.92	259.23	723.34
Profit													
*Paid-out cost* (USD ha − 1)	−	493.17	1046.35	−26.57	644.03	471.29	1041.32	−295.95	−17.80	353.15	592.03	−493.95	−218.43
*Paid-out + opportunity cost of family labor* (USD ha − 1)	−	310.01	863.19	−209.73	460.87	288.13	858.16	−479.11	−200.96	169.99	408.87	−677.11	−401.59
Benefit-cost ratio in terms of gross revenue													
*Paid-out cost* (USD ha − 1)	−	1.65	2.10	0.96	1.70	1.63	2.10	0.60	0.98	1.46	1.62	0.34	0.77
*Paid-out + opportunity cost of family labor* (USD ha − 1)	−	1.33	1.76	0.78	1.42	1.31	1.76	0.48	0.82	1.18	1.36	0.28	0.64

Source: Multi-locational trials. Notes: 1 USD = BDT 78.62 is used for computation (https://www.exchangerates.org.uk)], and include only the variable costs of maize production. In costs and returns column, items within parenthesis next to cost or return components in each cell denote corresponding units of measurement. High fertilizer treatments were as recommended by the Bangladesh Agricultural Research Council (BARC).

‘psd’=person day of work, i.e. 1 ps d = 8 h of work.

Fertilizer rates for the LF treatment were as follows:

^a)^ Urea (110.0 kg N ha-1), TSP (30.0 kg P ha-1), MOP (47.0 kg K ha-1), Gypsum (80.0 kg S ha-1), Zinc sulphate (0.0 kg Z ha-1) and Borax (0.0 kg B ha-1).

^b)^ Urea (100.0 kg N ha-1), TSP (36.0 kg P ha-1), MOP (56.0 kg K ha-1), Gypsum (45.0 kg S ha-1), Zinc sulphate (0.0 kg Z ha-1) and Borax (0.0 kg B ha-1).

^c)^ Urea (114.0 kg N ha-1), TSP (29.0 kg P ha-1), MOP (37.0 kg K ha-1), Gypsum (62.0 kg S ha-1), Zinc sulphate (3.0 kg Z ha-1) and Borax (0.7 kg B ha-1).

^d)^ “Glycel” at 3.7 ltr ha^−1^ and “Gramoxon” at 2.5 ltr ha^−1^ was applied in first and second years, respectively at all the sites.

^e)^ “Karate” at 0.75 ltr ha^−1^ was applied to control cut worm.

^f)^ Prevailing wage rates for male and female labors in the area were used to calculate the corresponding value of male and female family labor.

Considerable heterogeneity was observed with respect to fertilizer costs among sites, Low Fertilizer at Kalapara e.g., incurred US$ 135 ha^−1^, while Low Fertilizer at Patuakhali incurred the lowest cost for chemical fertilizer use (US$122 ha^−1^). Since irrigation had no consistent effect on maize yield (Fig. 7b), and in order to keep matters simple, we assumed an average cost of $97.51 ha^−1^ for all irrigation treatments for all years. The average gross return for maize production under Low and High Fertilizer treatments in the Barisal sites in 2016 was USD 1,250 ha^−1^ and USD 1,995 ha^−1^, respectively (Table 5). In 2017, yields were considerably lower. As a result, in Barisal, there was a 42% decline in gross return of Low Fertilizer (i.e. from USD 1250 ha^−1^ to USD 723 ha^−1^) in the second year as compared to the first. In the case of High Fertilizer, gross returns declined by only 22% (to USD 1560 from USD 1995 ha^−1^). In Patuakhali, gross returns from 2016 trials were USD 1,219 and USD 1,990 ha^−1^ for Low and High Fertilizer, respectively. Gross return however declined to USD 445 and USD 898 ha^−1^ in 2017 for the same treatments (i.e. they declined by almost 63% and 55% respectively). Maize grown at Kalapara in 2015/16 conversely achieved a gross return of USD 1,113 ha^−1^ under Low Fertilizer, while the gross return of the High Fertilizer treatment was USD 1,541 ha^−1^. This however declined substantially to USD 259 ha^−1^ and USD 723 ha^−1^, respectively for Low and High Fertilizer in 2016/17.

We analyzed the rate of return for maize from investment in high vs. low fertilizer rates. In non-saline environments of Barisal, the lower rate fetched an average 31% RoR across both years. The higher nutrient rate conversely had an RoR of 90%, indicating potential for substantially higher return for maize grown with recommended fertilizer rates in Barisal. In the moderately saline environments of Patuakhali, on average, the RoR from the Low Fertilizer treatment was 12%, compared to 54% for High Fertilizer. Closer to the coast, RoRs observed for Kalapara were -10% and 20%, respectively, for Low Fertilizer and High Fertilizer. Averaging the costs and returns over the two year study period, farmers utilizing recommended fertilizer rates could potentially achieve additional profits over paid-out costs from maize by USD 612, 424 and 257 ha^−1^, respectively over Low Fertilizer in Barisal, Patuakhali and Kalapara. Averaging the costs and returns for the two year period indicates that the increase in benefit cost ratios (BCR) with respect to paid-out costs for High Fertilizer in Barisal, Patuakhali and Kalapara are 46%, 38%, and 33% respectively, compared to Low Fertilizer.

## 4 Discussion and conclusions

This study examined irrigation scheduling and nutrient rates for maize and wheat in multi-year, multi-locational trials in marginal and saline environments of coastal Bangladesh.

The water tables remained close to the surface at all three locations. At Barisal, the measured depths never exceeded 2.75 m, while at the other two more southern sites, they tended to range between 1–2 m. Water table depths for the period of 2003–2007 at Patuakhali reported by Dalgliesh and Poulton (2011) tended to be about 0.5 m closer to the surface than our observations during the *rabi* season. Water table depths at the beginning of the measuring periods were similar for Kalapara and Patuakhali in all three years. Under dry conditions, the average daily decline ranged between 0.007 m (Barisal 2015) and 0.014 m (Kalapara 2015). The drop in the water table between January and March can be caused by lateral outflow, as well as capillary upflow into the rooting zone and to the soil surface.

Capillary upflow occurs because of the potential difference between the saturated soil layers and the drier upper layers. Based on data reported by Talsma (1963), Meyer (pers. comm) in (Wu et al., 1999) described an upflow function varying with soil texture. Conditions controlling upflow rates were further summarized and discussed by Ayars et al. (2006): Sandy clay loams and clay loams, i.e., the soils found in this study, are the ones from which upflow occurs from the lowest depths. An upflow of at least 1 mm day^−1^ can still be expected for depths of 6 m for these soils. Another main driver of upflow is the difference between soil water content of the unsaturated layer and lower limit of plant available water, i.e., a wet soil has higher upflow rate than a dry soil. Upflow rates are also controlled by the distance between the root zone and the water table. They decrease with distance between the two. Thus, the soils found in our study are highly conducive to high upflow rates from the water table, since the depth of the water table stayed mostly between 1–2 m and was constantly below 2 m in one site year only (Barisal 2015).

The water table rose quickly in response to high rainfall events late April and May 2015, May 2016 and the unseasonably heavy rains in March of 2017 at Kalapara, where a rainfall of 144 mm had occurred between March 8–11. Water table depths had been measured seven days before (March 1) and 11 days after the event (March 22). Between those dates, a raise in the water table by 0.68 m was measured, from 1.42 to 0.75 m depth. This sharp response can be attributed to the capillary fringe effect, where the water seeps up from a water table by capillary action and keeps the soil water content above DUL. Small amounts of water fill the capillary menisci and bring the water table to the surface. This is an accordance with Gillham (1984) and Miyazaki et al. (2012), who described the physical processes of a highly disproportionate response of the water table to precipitation events as the “reverse *Wieringermeer* effect”.

For the fields in our study, the proximity of the water table to the surface, as well as the presence of soils that have textures that are favorable for upflow, such as in the clay loam, silt loam and silty clay loam appear to create a favorable environment for a significant contribution to the water balance of the root zone. Accordingly, measured soil moisture content was generally above drained upper limit for depths below 0.5 m. The volumetric soil moisture data presented here are not representative for the dynamics that occurred during the entire growing season, since samples were taken only at sowing and then during the grain filling stage or at harvest. They nonetheless indicate that there was an ample supply of soil moisture, an observation backed by the limited irrigation treatment effect on yield. Irrigation had no effect on yield of wheat and a significant effect on yield of maize in 2016 only, the year which had the lowest amount of precipitation between January and April. For the wet treatment, the yield increase was 0.6 Mg ha^−1^ over the intermediate and 1.2 Mg ha^−1^ over the dry treatment. These results suggest that as long as the crop can be well established with a starter irrigation and a subsequent irrigation at V8 in maize and crown root initiation in wheat, a yield increase with additional irrigation is unlikely in our study region, except for dry years, as in 2016. In a study conducted near Shatkira, in the southwest of Bangladesh and approximately 250 km from our locations, Murad et al. (2018) reported a yield increase of 0.6 Mg ha^−1^ in 2016 and 0.9 Mg ha^−1^ in 2017 with the addition of a 3rd irrigation during grain filling of maize. That experiment, which was established on a single-location research station, consisted of plots sown approximately one month earlier than in the current study. Total observed rainfall was less than 10 mm in March and April of 2016 and 2017, whereas at our sites, BMD reported 260 mm and more during these months in 2017. The contrast in these precipitation data point to the wide variability in climatic conditions within central and western coastal Bangladesh (the latter tending to have deeper water tables and greater salinity). Hence, a dynamic, in-season advisory system for irrigation scheduling, taking into account actual weather conditions and forecasts, could be a useful tool for farmers. It could help farmers obtain optimal maize yields in dry winters.

Soil salinity was negligible (∼0.5 dS m^–1^) at Barisal. This is in agreement with Dalgliesh and Poulton (2011). The results for Patuakhali, which, according to SRDI (2000) has salinity levels that are below 2 dS m^–1^ (Fig. 1), indicated higher soil salinity than those reported by SRDI, at least for the wheat field. Its values at sowing and at harvest were in the range of 2.5 to 3 dS m^−1^. The last observation from the 2015/16 maize field was taken after harvest, after significant rainfall had occurred in May of 2016. As such, rainfall may have partially leached some salt from the root zone. For that reason, its salinity level probably remained below 2 dS m^−1^. At Kalapara, large differences in salinity levels between the wheat and maize field (Fig. 5, Fig. 6) were observed in the first year, despite of the fact that the fields were within 150 m of each other and were managed by the same farmer. Noteworthy is the accumulation of salt in the top layer (0 to 0.1 m depth) at that location. As compared to the layer below (0.1 to 0.2 m), its salinity levels were up to 2.5 dS m^–1^ higher for the wheat fields in both years and maize in the first year. This is in agreement with data from the Yellow River Delta, where Yu et al. (2014) observed higher salinity levels of up to 1 dS m^–1^ in the top 0.1 m of the soil as compared to lower depths. The elevated salinity levels in the Patuakhali region warrant proper consideration when opting for less salt tolerant crop species, including maize (cf. Ayers and Westcot, 1985). Moreover, the groundwater salinity at Patuakhali was also elevated. It was in the range of 2–4 dS m^−1^. These data are in agreement with observations reported by Mainuddin et al. (2014). Care must therefore be taken when irrigating salt intolerant crops, such as mung bean (the acreage of which is growing in coastal Bangladesh), so that the water table does not raise to the surface due to the above mentioned “reverse *Wieringermeer* effect”.

Out of the three experimental locations, Kalapara was most proximal to the coast. It consequently had the highest soil and groundwater salinity levels, likely causing yield reductions in maize. According to Ayers and Westcot (1985), yield reductions are to be expected if soil salinity levels exceed 1.7 dS m^−1^ for maize, whereas the critical threshold for wheat is 6.0 dS m^−1^. Its maize yields were lower than those in Barisal, the non-saline site, in all three years. For irrigation water salinity levels, the critical thresholds are 1.1 and 4.0 dS m^−1^ for maize and wheat, respectively.

Wheat yield at all sites was impacted by wheat blast and leaf blight in late February of 2016, during the grain filling phase, with significant negative effects on productivity. Compared to other sites, wheat at Barisal was sown last and therefore got affected by diseases at an earlier grain filling stage than at the other two locations. It consequently suffered the largest yield loss. It had the highest yield in the first experimental season and the lowest in the second year, explaining the highly significant (*P* <  0.001) location by year interaction.

Given increasing labor scarcity issues in Bangladesh (Mottaleb et al., 2016), the lack of appropriate mechanization appears to be a bottle-neck for profitable maize production. Maize requires a considerable investment in labor, as its cultivation, apart from sowing, is generally not mechanized. Farmers spent about 86 person-days to cultivate 1 ha of maize, similar to reports by Gathala et al. (2015; 2016) for maize in northern Bangladesh under conventional tillage and hand planting regimes. When family labor is accounted for, the cost of maize cultivation increased by 19–25% in all the trial sites, for all fertilizer treatments. A similar observation was made by Bhattacharya et al. (2019) for maize in southern Bangladesh, where they detected a 25–29% cost decline when the family labor (that accounted for 50% of the total labor) cost was not imputed.

Our findings suggest that a higher (recommended) fertilizer rate is associated with improved economic returns when compared to lower rates more representative of farmers’ current practices in all of the three locations studied. Similar findings have been noted by a number of other studies including of on-station (Maman et al., 2018) and on-farm (Witt et al., 2006) trials, computer simulation models (Donner and Kucharik, 2003), and economic analyses using survey data (Xu et al., 2006; Maine et al., 2007). Several studies favour balanced fertilizer use for higher profits in markets with stable prices (Darko et al., 2016; Liverpool-Tasie et al., 2017). In addition, due to differences in biophysical and prevailing local market conditions, economic returns were dissimilar across the three geographical locations in the study. A higher rate of return for fertilizers was found in the non-saline trial site in Barisal in the South-central part of the delta, with diminishing returns found in higher saline trial locations at Patuakhali and Kalapara towards the coast. Munns and Gilliham (2015) reported on the economic constraints associated with soil salinity including reduced profitability. Our data also corroborate other findings of smallholder farmers including an econometric analysis of maize in Burkina Faso (Theriault et al., 2018) and an experimental study in the Mekong Delta (Witt et al., 2006). The latter study suggested that the economic incentives for fertilizer use varied depending on prevailing biophysical circumstances in locations in which farmers grew their crops. The negative rate of return for the lower fertilizer rate in maize in Kalapara indicates chances of heavy yield losses where farmers are unable to invest in or access recommended rates of fertilizers. Majumdar et al. (2012) and Jat et al. (2012) also reported on the significant impact of fertilizer use, indicating the importance of educating prospective maize farmers on matching their yield targets with appropriate nutrient additions and rates. It should nonetheless be noted that maize cultivation under recommended management can also yield poorly in unfavourable weather conditions. For instance, maize produced in 2017 incurred heavy yield losses in our trial locations presumably due to unseasonal, excessive rainfall and waterlogging in March. The resulting BCR for High Fertilizer at Kalapara was 0.77, indicating a negative return. Initiatives to diversify cropping systems and include maize as a viable winter season crop in the central coastal region of Bangladesh will therefore require efforts to identify and target appropriate environmental conditions, matched with extension initiatives educating farmers on ‘best-bet’ and low-risk agronomic and financial management, and last but not least, access to credits at fair conditions.

## Figures and Tables

**Table:** 

				Sand	Silt	Clay	Bulk Density
Village	Depth (m)	pH	OM (%)	(g/g)			Mg/m3
Char Baria	0 – 0.1	7.61	2.49	0.20	0.47	0.34	1.30
	0.1 – 0.2	8.29	1.16	0.17	0.48	0.36	1.62
	0.2 – 0.3	8.39	0.91	0.19	0.51	0.30	1.55
	0.3 – 0.4	8.44	1.17	0.26	0.49	0.25	1.54
	0.4 – 0.5	8.47	0.72	0.25	0.58	0.17	1.52
	0.5 – 0.6	8.47	0.97	0.33	0.50	0.17	1.49
	0.6 – 0.7	8.42	0.80	0.19	0.67	0.14	1.47
	0.7 – 0.8	8.42	0.74	0.16	0.71	0.12	1.44
	0.8 – 0.9	8.45	1.23	0.14	0.69	0.14	1.44
	0.9 – 1.0	8.35	0.76	0.18	0.72	0.10	1.43

Sayestabad	0 – 0.1	8.40	2.00	0.03	0.66	0.30	1.38
	0.1 – 0.2	9.17	1.48	0.04	0.65	0.31	1.64
	0.2 – 0.3	9.53	1.44	0.04	0.65	0.30	1.60
	0.3 – 0.4	9.67	1.33	0.03	0.62	0.35	1.59
	0.4 – 0.5	9.57	1.33	0.03	0.54	0.43	1.58
	0.5 – 0.6	9.60	1.15	0.06	0.67	0.27	1.54
	0.6 – 0.7	9.53	0.72	0.05	0.70	0.25	1.54
	0.7 – 0.8	9.57	0.96	0.05	0.68	0.27	1.56
	0.8 – 0.9	9.60	0.48	0.04	0.69	0.27	1.56
	0.9 – 1.0	9.70	0.77	0.04	0.64	0.32	1.56

Badarpur	0 – 0.1	6.37	2.13	0.03	0.70	0.27	1.45
	0.2 – 0.2	7.23	1.85	0.04	0.66	0.30	1.54
	0.2 – 0.3	7.73	2.07	0.03	0.69	0.28	1.56
	0.3 – 0.4	7.73	1.92	0.04	0.59	0.38	1.59
	0.4 – 0.5	7.70	1.80	0.01	0.47	0.52	1.56
	0.5 – 0.6	7.67	1.74	0.06	0.47	0.46	1.59
	0.6 – 0.7	7.67	1.11	0.03	0.44	0.53	1.51
	0.7 – 0.8	7.67	1.62	0.02	0.53	0.44	1.50
	0.8 – 0.9	7.63	1.35	0.04	0.63	0.33	1.49
	0.9 – 1.0	7.50	1.19	0.03	0.76	0.21	1.51

Jainkathi	0 – 0.1	5.85	1.23	0.02	0.65	0.33	1.29
	0.3 – 0.2	6.87	1.20	0.01	0.66	0.32	1.51
	0.2 – 0.3	7.65	1.09	0.02	0.68	0.30	1.53
	0.3 – 0.4	7.31	1.32	0.02	0.62	0.36	1.51
	0.4 – 0.5	7.31	0.87	0.01	0.61	0.38	1.44
	0.5 – 0.6	7.35	0.52	0.03	0.52	0.42	1.38
	0.6 – 0.7	7.26	0.72	0.03	0.52	0.46	1.36
	0.7 – 0.8	7.33	1.16	0.03	0.51	0.46	1.34
	0.8 – 0.9	7.34	0.62	0.03	0.63	0.34	1.41
	0.9 – 1.0	7.35	0.57	0.02	0.66	0.33	1.40

Kalapara	0 – 0.1	5.43	1.83	0.03	0.73	0.25	1.29
	0.4 – 0.2	7.05	1.30	0.03	0.71	0.26	1.60
	0.2 – 0.3	7.80	1.63	0.06	0.70	0.24	1.47
	0.3 – 0.4	7.88	1.48	0.08	0.69	0.23	1.44
	0.4 – 0.5	7.89	0.73	0.06	0.72	0.22	1.41
	0.5 – 0.6	7.54	1.73	0.08	0.69	0.24	1.44
	0.6 – 0.7	7.97	1.51	0.05	0.73	0.22	1.45
	0.7 – 0.8	7.98	1.73	0.02	0.76	0.22	1.45
	0.8 – 0.9	7.65	1.43	0.04	0.70	0.27	1.43
	0.9 – 1.0	8.01	1.43	0.05	0.74	0.21	1.44

**Table:** 
